# Title 1 Changes: Bringing the Older Americans Act Definitions to 2020

**DOI:** 10.1093/geroni/igaa057.2517

**Published:** 2020-12-16

**Authors:** Thomas Eagen

**Affiliations:** University of Washington, Washington, District of Columbia, United States

## Abstract

This session will review the changes to Title I of the Reauthorization of the Older Americans Act. This title provides important updates and modernization to the definitions used throughout the Older Americans Act. Some definitions were modified such as adding the prevention of sexually transmitted diseases, chronic pain management, and screening for suicide risk to evidence-based health promotion programs under the definition of disease prevention and health promotion services. Other changes include services to provide screening for fall-related injuries, services that are responses to public health emergencies, and services to prevent and address negative health effects associated with social isolation. Title I also includes a series of important including access to person-centered services and coordinating services provided by aging and disability resource centers. Several other changes to Title I will be discussed.

